# Radiomics Applications in Head and Neck Tumor Imaging: A Narrative Review

**DOI:** 10.3390/cancers15041174

**Published:** 2023-02-12

**Authors:** Mario Tortora, Laura Gemini, Alessandra Scaravilli, Lorenzo Ugga, Andrea Ponsiglione, Arnaldo Stanzione, Felice D’Arco, Gennaro D’Anna, Renato Cuocolo

**Affiliations:** 1 Department of Advanced Biomedical Sciences, University of Naples “Federico II”, 80131 Naples, Italy; 2 Department of Radiology, Neuroradiology Unit, Great Ormond Street Hospital, London WC1N 3JH, UK; 3 Neuroimaging Unit, ASST Ovest Milanese, 20025 Legnano, Italy; 4 Department of Medicine, Surgery and Dentistry, University of Salerno, 84081 Baronissi, Italy

**Keywords:** head and neck tumors, radiomics, diagnostic imaging, artificial intelligence

## Abstract

**Simple Summary:**

Head and neck tumors (HNTs) are associated with a high mortality due to their commonly insidious and asymptomatic development. Regarding risk stratification and long-term patient outcome prediction, routine clinical evaluation by radiologists has several limitations. Numerous researchers have assessed the usefulness of radiomics and artificial intelligence in the context of head and neck tumor imaging given the exponential development of these technologies in medical imaging. These were geared at the creation of reliable and reproducible models based on quantitative data. Even if there are still a few obstacles to their widespread usage in clinical practice, it is clear that they have the potential to be revolutionary. In this paper, we provide a thorough overview of radiomics and artificial intelligence applications in head and neck tumor imaging.

**Abstract:**

Recent advances in machine learning and artificial intelligence technology have ensured automated evaluation of medical images. As a result, quantifiable diagnostic and prognostic biomarkers have been created. We discuss radiomics applications for the head and neck region in this paper. Molecular characterization, categorization, prognosis and therapy recommendation are given special consideration. In a narrative manner, we outline the fundamental technological principles, the overall idea and usual workflow of radiomic analysis and what seem to be the present and potential challenges in normal clinical practice. Clinical oncology intends for all of this to ensure informed decision support for personalized and useful cancer treatment. Head and neck cancers present a unique set of diagnostic and therapeutic challenges. These challenges are brought on by the complicated anatomy and heterogeneity of the area under investigation. Radiomics has the potential to address these barriers. Future research must be interdisciplinary and focus on the study of certain oncologic functions and outcomes, with external validation and multi-institutional cooperation in order to achieve this.

## 1. Introduction

Head and neck tumors (HNTs) account for over 830,000 cases annually worldwide [1] and are associated with a high mortality due to their commonly insidious and asymptomatic development. This often leads to a late diagnosis of disease, in more advanced stages. HNTs comprise a large spectrum of tumors and tumor-like conditions [2], which can arise from different tissues included in this anatomical region, such as paranasal sinuses, pharynx, oral cavity, larynx, thyroid, lymph nodes and associated soft tissues and bones. Squamous cell carcinomas (SCCs) represent the most common histological type, accounting for 90% of all HNTs [3]. Risk factors include tobacco and alcohol use [4] as well as, more recently identified, human papillomavirus (HPV) infection [5]. The latter represents the main cause of an increasing incidence of head and neck squamous cell carcinoma (HNSCC) in the USA [6], mainly consisting of oropharyngeal squamous cell carcinoma (OPSCC) and nasopharyngeal carcinoma (NPC). Among HNSCCs, NPC represents a peculiar entity with a distinct epidemiology. It is a rare malignant epithelial tumor [7] arising from the superior region of the pharynx’s mucosa, usually from the lateral pharyngeal recess (i.e., Rosenmüller’s fossa) [8], with evidence of squamous differentiation. An interplay of different causes is involved in the etiology of this disease, including genetic, viral and environmental risk factors, such as nitrosamine-containing food consumption and smoking. Its strong association with the Epstein–Barr virus infection makes the incidence of NPC significantly higher in some endemic regions, including southeast Asia and China [9]. According to the 2017 World Health Organization (WHO) Classification of HNTs, NPCs are classified into three subtypes, non-keratinizing (NK-NPC), keratinizing (K-NPC) and basaloid [10], as also confirmed in the recent 5th edition of the WHO Classification. NK-NPC represents the most common histologic subtype. Magnetic resonance imaging (MRI) is the modality of choice for diagnosis, staging and in the evaluation of response to treatment in HNTs, as its high soft tissue contrast allows a more accurate delineation of tumor margins compared to computed tomography (CT) and spectral photon-counting CT [11]. Furthermore, the emerging field of radiomics has shown that a large amount of additional quantitative information, otherwise undetectable, can be extracted from medical images of these patients.

**Figure 1:**
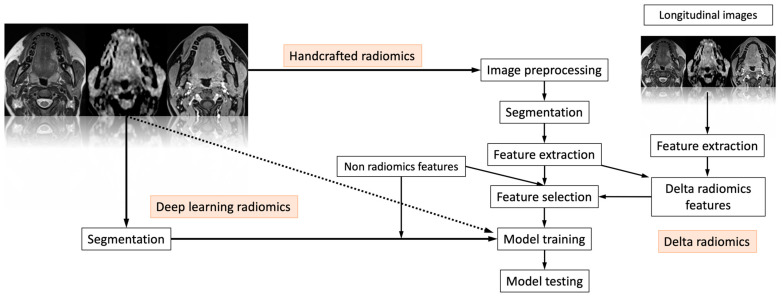
The “radiomic workflow” involves a series of iterative steps for reproducible and consistent extraction of imaging data. These steps include image acquisition, feature extraction and feature selection. This may be possible through deep-learning radiomics, handcrafted radiomics and delta radiomics. Finally, the selected features are used to test the final model.

Radiomics is a quantitative method of approaching medical imaging, which seeks to augment the data already available to doctors through sophisticated mathematical analysis. Using analytical techniques, radiomics quantifies textural information by mathematically extracting the spatial distribution of signal intensities and pixel inter-relationships [12]. Numerous imaging studies from different fields have already been published using this approach. The use of radiomics in the study of the head and neck region, particularly for neoplastic pathology, is one of the newer use areas. This is achievable through the characterization of pixel gray level distribution patterns, which can then be analyzed by machine-learning (ML) algorithms, potentially providing information on tumor physiology, which could have an important impact on the management and improve prognosis of these tumors in the near future. Figure 1 shows a classic “radiomic workflow” involving a series of steps for reproducible and consistent extraction of imaging data. These steps include image acquisition, feature extraction and feature selection. This may be possible through deep-learning (DL) radiomics, handcrafted radiomics and delta radiomics.

Based on how images are converted into data that can be mined, radiomics has two primary branches: “deep-learning” and “handcrafted radiomics”. In contrast to deep learning, which uses complex networks to “extract and analyze” its own features, handcrafted features are obtained by formulae that are mostly based on intensity histograms, shape attributes and texture matrices, which can be used to identify phenotypical properties of the radiological image [13]. Delta radiomics is the study of characteristics throughout time and how they change in order to predict a patient’s response to treatments [14]. Finally, the selected features are used to test the final model.

**Figure 2:**
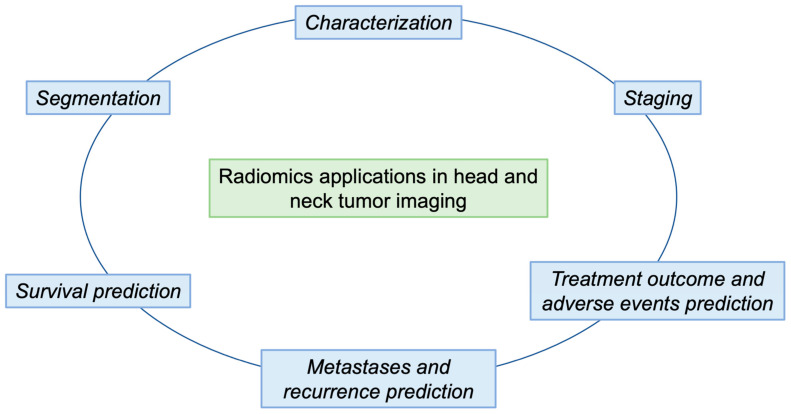
The outline of our paper is shown in Figure 2. In particular, we describe the main steps of a radiomic workflow (segmentation and characterization) in order to obtain predictions of survival, metastasis and recurrence, and treatment responses.

In this review, we will provide an overview of radiomics and machine-learning studies, focusing on the different research areas in which these techniques can be implemented in relation to HNTs, such as lesion segmentation, grading, differential diagnosis, prediction of prognosis, evaluation of treatment response and prediction (Figure 2). Table 1 and Table 2 present a summary of the studies discussed in the text, arranged by subtopic.

## 2. Segmentation

Grouping portions of a picture that belong to the same class of objects is known as segmentation. Because it establishes a tumor’s region of interest (ROI), from which imaging data are collected and processed into machine-readable quantitative attributes, segmentation is essential to the creation of a radiomic workflow. Depending on the approach used, the tumor lesion may be delineated as a two-dimensional or three-dimensional ROI or volume of interest (VOI), respectively [77]. Segmentation can be performed with different methods: manual, semi-automatic and automatic. There are advantages and disadvantages of each method [78]. Precision definitions of ROIs and/or VOIs are possible with manual segmentation using a mouse or a graphic tablet, especially when trained radiologists apply it to small datasets. However, this method involves time-consuming procedures and may be subject to high intra- and inter-observer variability, resulting in bias in radiomic pipeline results. Applying algorithms that make use of various image delineation strategies, such as region-growing, level set, graph cut and active contour (snake) techniques, is the process of semi-automatic segmentation [79]. Despite the fact that this technique reduces labor tasks and improves radiomic feature robustness [15], the stability of radiomic models remains susceptible to subjective bias, especially in cases of intensive user correction. Medical image segmentation has recently used a completely automated technique. In the identification and segmentation of lesions, it has shown excellent results, and it has also eliminated potential intra- and inter-observer differences [78]. Large data requirements and the generalizability of the taught algorithms are the key drawbacks [79]. On the one hand, these approaches could help reduce the workload and increase reproducibility in craft radiomic research. On the other hand, they do not need picture segmentation for classification. Given the absence of standardized segmentation techniques, which might result in inconsistently replicable models, tumor segmentation presents a significant barrier to the robustness of radiomic characteristics, particularly for manual and semi-automated approaches [15]. Indeed, emerging exploratory work has been aimed at assessing the extent to which the stability of radiomic features may be affected by segmentation variability [17]. Texture analysis has also been applied to create automated segmentation models. Using radiomics to distinguish between normal and pathologic tissue in HNTs, Yu et al. developed a multivariate model, which could identify pathological pixels using a combination of positron emission tomography (PET) and CT gray-tone difference features [18]. Using this approach, a co-registered multimodality pattern analysis segmentation system (COMPASS) has been developed to identify the radiation therapy target using PET and CT images. This is able to identify the tumoral area with results comparable to manual segmentation by expert radiation oncologists [19], possibly reducing inter-observer variability and improving treatment planning accuracy. In a recently published paper, Prezioso et al. provide another example of automatic segmentation of head and neck lesions using a DL-based framework for automatic segmentation of salivary gland tumors [80].

## 3. Characterization

Recent radiomics research works have demonstrated the correlation between bio-imaging traits (human papilloma virus (HPV) status, somatic mutations, methylation, subtypes of gene expression and PD-L1 protein expression levels) in HNSCC. The subject that has generated the most attention among them is HPV status. Younger patient age at presentation, unique tumor morphology (smaller original tumors, significant cervical adenopathy) and a better response to radiation treatment are all related to the virus’ existence [81]. If supported by sufficient evidence, radiomics-based biomarkers could be used in the future as a viable alternative to confirm HPV status after positive p16 immunohistochemical tests [81]. Several studies have been conducted to define HPV status in HNSCC using texture analysis. To date, the majority of these have investigated the value of CT-based radiomics. For example, both Buch et al. and Fujita et al. examined the association of individual texture features with HPV status. The first research group identified three features (histogram-derived median and entropy and gray-level co-occurrence matrix (GLCM) entropy) on contrast-enhanced CT images of primary oropharyngeal squamous cell carcinoma, which showed statistically significant differences in relation to patient HPV status [20]. Similarly, Fujita et al. were able to identify 16 texture parameters with the potential to distinguish HPV status in non-oropharyngeal carcinoma [21]. Regarding other imaging modalities, Vallieres et al. reported on the value of Fludeoxyglucose (FDG)-PET features as HPV status biomarkers when used in combination with different machine-learning algorithms [22]. With regard to MRI-based biomarkers, some studies assessed that MRI radiomics models could be used in future as an effective imaging biomarker to confirm HPV status after positive p16 immunohistochemical tests [81]. Differences in the apparent diffusion coefficient (ADC) value have been reported in some previous MRI-based studies [23,24,82]. Moreover, Marzi et al. obtained good results in differentiating HPV status in OPSCC using a multifactorial model incorporating diffusion-weighted imaging (DWI) and clinical features. They extracted first- and second-order radiomic features from ADC maps and trained different machine-learning algorithms from that dataset [25]. Chong Hyun Suh et al. conducted a retrospective study in 60 patients with new histological diagnosis of OPSCC. They manually delineated the tumor area in four sequences (axial T1 weighted images (WI), fat-suppressed T2 WI, axial fat-suppressed contrast-enhanced T1 WI and ADC maps from DWI) and then demonstrated that three machine-learning classifiers (logistic regression, random forest and XGboost) trained with quantitative radiomics features extracted from those variously combined sequences were accurate in predicting HPV status [26]. Additionally, Sohn et al. developed a model for diagnosing HPV status in patients with oropharyngeal cancer using six MRI radiomic features (post-contrast 3D T1WI and T2 WI sequences) [27]. Beyond HPV status, the use of radiomics biomarkers has also been proposed to identify HNSCC molecular subtypes in several studies. Aerts et al. [28] associated three radiomics features with the presence of mutations in as many driver genes (TP53, FAT1 and KMT2D) and found that FAT1 had a significant association with all of them [29]. Huang et al. [30] studied several molecular HNSCC “phenotypes” (five DNA methylation subtypes, four previously identified HNSCC gene expression subtypes and five common somatic gene mutations) and considered 540 CT radiomics features extracted from pretreatment scans of 113 patients. Zhu et al. [31] reported a correlation between radiomic features extracted from contrast-enhanced CT images and genome data in a public cohort of 126 HNSCC patients, identifying over 5000 statistically significant associations. Interestingly, Chen et al. [32] reported a significant association between FDG PET textural features and expression of PD-L1, which correlate with response to PD-1 blockers, such as nivolumab or pembrolizumab [83], in patients with oropharyngeal and hypopharyngeal SCC. In the field of preoperative stratification of thyroid tumors, an algorithm that used linear discriminant analysis focused on DWI and ADC data was proposed. The authors report a greater performance of textural features in differentiating between benign and malign lesions (area under the curve (AUC) = 0.97, sensitivity = 92%, specificity = 96%) compared to ADC alone (AUC = 0.73, sensitivity = 70%, specificity = 63%) [33]. Jansen et al. analyzed the parametric maps of K^trans^ and V_e_, which are indices of tumor vascularity, in HNSCC patients before and during the treatment, obtained with dynamic contrast-enhanced perfusion imaging. They showed a significantly higher energy feature in the scans performed during the treatment, suggesting that texture analysis could be used together with standard MRI perfusion maps to provide additional information in head and neck oncological patients [34,35,36]. In addition, some studies aim to predict p53 status, as a positive status is associated with poor prognosis [84,85,86]. Dang et al. showed that MRI texture analysis could predict p53 status in oropharyngeal squamous cell carcinoma with an accuracy of 81.3% (*p* < 0.05). The variables that stood out significantly were those thought to be due to differences in vascularity between p53(+) and p53(−) status [37].

## 4. Staging

Pre-treatment staging is an important point in diagnosis and therapeutic planning, as well as a factor closely related to tumor prognosis. The main treatment is surgery, but there are also several treatment options, including induction chemotherapy, concomitant chemoradiotherapy, targeted therapy or immunotherapy [87,88]. Studies have shown that the T-stage of head and neck tumors and lymph node status greatly influence the treatment choice, and thus, the prognosis of cancer patients [89,90]. Prior to therapy, a reliable radiomics evaluation of the tumor’s stage can assist direct treatment decisions, ensuring lower risk of adverse effects and recurrence. Radiomics could be used to successfully establish a T-staging model of locally progressed laryngeal cancer [88]. In particular, MRI radiomic signature was shown to be a supplemental tool for preoperative staging, differentiating stage III–IV from stage I–II squamous cell carcinoma [89]. Romeo et al. [40] predicted tumor grade and lymph node status (N) in squamous cell carcinoma of the oropharynx and oral cavity using a radiomic approach based on contrast-enhanced CT images. The determination of extra-nodal extension (ENE) of the tumor is important, since it represents an unfavorable prognostic factor and is associated with a higher risk of developing recurrent disease [91], as will be discussed in the next sections. Finally, a prospective study of a cohort of 96 patients with papillary thyroid carcinoma (PTC) enrolled patients who underwent neck MRI and subsequent thyroidectomy during the study interval. Aggressive and non-aggressive cancers can be distinguished using machine-learning MRI-based prediction algorithms. This is crucial before surgery, since it makes it easier to create individualized treatment strategies [91].

## 5. Treatment

Leaving out the prediction of surgical treatment outcomes (which can be predicted by the surgeon based on planned resection according to current guidelines), it would be appropriate to focus on the predictive ability of radiomics models regarding radiotherapy and chemotherapy outcomes [42].

### 5.1. Pre- and Intra-Treatment Imaging

Oncologists can devise individualized treatment plans and implement preventative measures to enhance therapy outcome and patient’s tolerance to therapy.

During radiation therapy (RT) for NSCLC, features computed from pre-treatment and weekly intra-treatment CT alter dramatically [42].

Cone beam CT (CBCT) systems may be able to conduct delta radiomics for image-guided radiation, enabling extensive research on tumor response to total dose, fractionation and fraction dosage. It has been demonstrated that repeatable characteristics from CBCT are just as effective in predicting overall survival in NSCLC patients as features from CT [92]. However, studies on CBCT delta radiomics are still only capable of evaluating repeatability and feasibility.

During pre- and intra-treatment evaluations, the preferred MRI sequences differed across investigations. The sequences selected vary, though; for instance, some authors utilize DCE-MRI to integrate pharmacokinetic modeling [34], while others employ DWI to increase the precision of lesion stratification [33]. The repeatability of pictures and, by extension, the textural qualities obtained from them may vary between MRI modalities in addition to sequence selection due to differences in scanner features. The possibility of bias from characteristics taken from a single sequence can be decreased using multiparametric techniques [43].

### 5.2. Short-Term Outcome and Adverse Events

A few studies aiming to estimate early response to induction chemotherapy and chemoradiotherapy (CRT) [44,45] in nasopharyngeal carcinoma have been conducted. In addition, it might also be useful to predict outcomes in adjacent non-cancerous tissues, such as glandular tissues (parotid and major salivary glands). A general decrease in parotid tissue complexity and heterogeneity has been observed in the literature [90]. The change in mean volume and intensity was found to be correlated with pre-treatment dosimetric parameters, suggesting a relationship between the dose schedule and estimated structural change after radiotherapy [93].

### 5.3. Long-Term Outcome and Adverse Events

CRT represents a usual treatment regimen [91]; however, adverse symptoms are occasionally seen, even in the long term. These include hearing loss, trismus and xerostomia. Radiation xerostomia is a common side effect and poses a challenge in the long-term management of patients [46,94]. A number of studies with heterogeneous endpoints have been performed in this regard: Sheikh et al. [47] predicted a binary endpoint of xerostomia at 3 months after radiation therapy; Liu et al. [48] applied regression analysis for the prediction of acute xerostomia; van Dijk et al. [49,50] used three different imaging modalities (CT, MRI, FDG-PET) for the classification of the binary outcome of long-term xerostomia. Although these results appear promising, their clinical application is limited due to lack of external validation, heterogeneity in image processing, statistical analysis and treatment outcome measures. Trismus may result from involvement of masticatory muscles in radiation therapy treatment fields, surgery or tumor invasion into mastication structures, or neural innervation of masticatory muscles [95]. Thor et al. [51] compared 24 imaging features extracted from post-contrast T1 WI sequences of four masticatory muscles in 10 patients with radiation-induced trismus after treatment with 10 control subjects. The medial pterygoid muscle was shown to have the greatest radiomic predictor discriminative capacity. The outcome is not statistically significant, but it may be a hint of how well radiomic biomarkers can predict post-radiation trismus. Cochlear radiomics may be used to anticipate hearing loss brought on by chemotherapy and radiation therapy, according to Abdollahi et al. They showed that the combination of radiomic features with clinical and dosimetric variables can predict radiotherapy-induced neural sensory hearing loss.

In the context of long-term outcome, we reserve the right to address “metastases and recurrence” and “survival” separately in the following sections.

## 6. Metastases and Recurrence

ENE of cervical lymph node metastases is an adverse prognostic feature linked to a higher probability of recurrent illness. This supports the use of chemotherapy in combination with adjuvant radiotherapy [40]. In individuals who are likely to need adjuvant chemoradiation, the detection of ENE might assist direct treatment decisions, lower morbidity and prevent surgery. Quantitative imaging methods were created and validated by Kann et al. for the identification of ENE prior to surgery [51,95]. On contrast-enhanced CT images, they segmented more than 600 lymph nodes and extracted 99 radiomic characteristics. These provided AUC values for ENE identification and lymph node metastases detection close to 0.9 by training ML classifiers. These results highlight the potential for quantitative imaging to enhance radiologist’s performance and guide the treatment of HNSCC. Zhang et al. [55] developed a model for pre-treatment risk assessment of distant metastasis in patients with nasopharyngeal carcinoma using MRI. They extracted 2780 radiomic features, among which 7 were selected to build a logistic regression model to classify patients at low or high risk of distant metastasis. They trained the model using a retrospective cohort of 123 untreated patients with non-metastatic status (AUC 5.827) and validated the trained model using an independent retrospective cohort of 53 patients (AUC 5.792). Other studies suggest the use of MRI, CT and/or PET imaging radiomics and ML to predict tumor recurrence after radiotherapy and/or chemotherapy for several HNTs [56,57,58]. Through the study of a large dataset of pre-treatment contrast-enhanced CT scans (465 cases of oropharyngeal squamous cell cancer), a model capable of significantly discriminating between high and low probability of recurrence groups was analyzed by the M.D. Anderson Cancer Center’s Quantitative Head and Neck Imaging Working Group [59]. Finally, in relation to recurrence, it was possible, through the selection of eleven imaging features, to construct a radiomic score (Rad-score) capable of predicting local recurrence-free survival (LRFS). Rad-scores were generated using Cox’s proportional hazards regression model and can reliably predict LRFS in patients with non-metastatic T4 stage [60].

## 7. Survival

With the development of medical diagnosis and treatment technology, great progress has been made in the treatment of HNTs [96,97,98]. However, at the time of first diagnosis, many patients are already in an advanced stage of disease. With five-year survival rates ranging from 25% for hypopharyngeal carcinoma (HPC) to 80% for nasopharyngeal carcinoma (NPC), the prognosis is still poor [61,62]. In order to create even more correct treatment programs, it is necessary to predict patients’ survival rates more precisely.

In research reports on the use of radiomics in HNC, radiomic models related to survival prediction are the most numerous. Shen et al. [61] sought to explore the predictive value of the radiomic model based on MRI features. Out of 327 patients, they established five models. The prognostic performance of these models was evaluated by Harrell’s concordance index (C-index). It was found that the best model was the one incorporating radiomics, global health stage and DNA in non-metastatic tumors. Yuan et al. [62] found that radiomic signature based on MRI is an independent prognostic factor for patients with HNSCC, as also highlighted by another study [63]. In addition, others have used pre- and post-operative PET/CT radiomic features for HNSCC and found that combining clinico-pathological features with pre- and post-treatment PET/CT radiomic features can substantially improve the prediction of overall survival (OS) of HNSCC patients [64,65]. Zhai et al. [66], using 240 contrast-enhanced CT data, reported significantly better prognostic performance with a combined model than a model based on clinical variables alone for disease-free survival in HNSCC. Using 542 cases of oropharyngeal SCC from Canada, Leijenaar et al. [67] validated a radiomics model previously devised by Aerts et al. [28] on 422 cases, which showed significant prognostic differentiation in Kaplan–Meier analysis of OS in all sub-cohorts. Radiomics-based outcome prediction used CT with and without contrast, T1WI and T2WI MRI sequences (with contrast) and FDG-PET, as well as DWI [45], 18F-fluorothymidine PET [99] and perfusion CT [68]. Most of the studies applied multivariate Cox proportional hazard models. The performance, expressed by the hazard ratio of the Cox model, and the accuracy, expressed by the C-index, of the radiomic or combined models were superior to the clinical models with respect to the prediction of various outcomes. An investigation by Parmar et al. [63], analyzing features extracted from pre-treatment CT images of four independent cohorts of HNTs (878 patients in total), showed that radiomic clusters are significantly associated with patient survival and tumor stage. Parmar et al. analyzed 13 feature selection methods and 11 machine-learning classification methods chosen for simplicity, efficiency and popularity in the literature. Specifically, they identified three classifiers and feature selection methods that demonstrated high performance and stability in predicting 3-year OS in head and neck cancer, suggesting that these machine-learning methods should be the starting point for radiomics-based prognostic analyses due to their consistency. El Naqa et al. [100] examined the characteristics of pre-treatment PET images of nine HNT patients. Using the most predictive features, they were able to construct a two-metric model predicting OS with an AUC of 1. In a retrospective study of 72 patients using 2D CT texture analysis, textural features were found to be associated with OS in patients with locally advanced HNSCC treated with induction chemotherapy [69]. In addition, texture analysis of CT, PET or MR images before treatment has been used to predict progression-free survival or OS in several HNTs of the mucosa or thyroid [70,71,72,73,74,75]. Leijenaar et al., using contrast-enhanced CT radiomic features, assessed that p16 and the radiomics-based classifier had the same potential to differentiate the risk of patients in the survival curve using Kaplan–Maier analysis [76].

## 8. Limitations of Radiomics

### 8.1. Current Issues

Radiomic analyses are very promising in assessing several characteristics of head and neck malignancies, but there are a number of limitations that must also be considered [77]. The majority of recent radiomics research works have a retrospective, monocentric design, which advocates for caution in interpreting the reported findings. In particular, the small sample size frequently characterizing these works may lead to a patient selection bias, not accurately reflecting the overall population [101]. Furthermore, few device manufacturers and data acquisition techniques are often employed, and this could also result in random patterns that add biases into the models. Unfortunately, these are difficult to identify without access to larger and more varied datasets. In general, these issues may lead to models that cannot duplicate their performance in new research trials. The low level of uniformity of radiological imaging protocols, which may also have an impact on the generalizability of the models and, consequently, their clinical application, represents another issue somewhat related to the latter [102]. Another problem is the extremely frequent lack of external validation, which may cause the predictive model to overfit. Extreme variability in the segmentation, feature extraction and selection, as well as the adopted modeling procedures still represent further drawbacks. In general, rather than the underlying biological lesion features, all the aforementioned issues constitute a possible source of unwelcome heterogeneity brought on by the traits of the patient cohort and imaging data. Instead of identifying this heterogeneity as the noise it actually is and dismissing it, radiomics pipelines run the risk of seeing it as a source of information and including it in the model’s classification process. Future works should therefore focus on radiomics pipeline standardization and prospective multicenter trial designs. Despite being in its early stages, efforts are being made to increase awareness of the methodological problems affecting current radiomics research, to encourage editors and reviewers to focus more on the technical details and clinical applicability of this work, to educate potential customers about commercial solutions based on radiomics and to gather curated, open medical images [103].

### 8.2. Potential Solutions

Briefly, the main limitations of radiomics are due to bias in three main steps: data collection and handling; model development; performance metrics. To lessen such bias, it is essential to be aware of it [104,105,106].

Careful data collection is critical to ML model development. Estimating the types, features and sizes of the data required is essential for locating and gathering the right datasets. First, a comprehensive study of the literature that incorporates advice from medical experts aids in the task [107]. The minimal dataset size required to demonstrate an effect and guarantee the brilliance of the trained model may be determined using statistical power estimate approaches and knowledge of similarly created ML models [108].

Training a heterogeneous model can help machine-learning systems perform better [109]. To this goal, data collection from several institutions with various patient compositions is beneficial. This issue can be improved thanks to the development of data de-identification technologies, federated learning and cloud data storage. The second strategy is to obtain information from many suppliers (such as imaging equipment or electronic medical records) while staying within the same organization. It might be beneficial to amass many brands and models, even older ones. Using open datasets is the third strategy [106].

Regarding model development, frequent bias is caused by overfitting. Early stopping, which tracks the model’s performance on the validation set and halts training when the validation measure drops or its validation loss rises over a few steps, is one method to lessen overfitting [110]. Model capacity reduction is another strategy for reducing overfitting. Fewer parameters limit the network’s ability to learn erroneous characteristics, pushing it to focus on learning the most crucial ones [111]. Regularization is another strategy to lessen overfitting. Regularization techniques include dropout layers and regularizations. Ensemble modeling might also help address overfitting [112]. The risk of overfitting is reduced by oversampling and undersampling, which prevent the model from seeing significantly more instances of one class than others during training [113].

Finally, attention must be paid to performance metrics to minimize bias [105]. Predictive models for head and neck cancer have been developed using twelve distinct classifiers [63]. A multi-classifier model that makes the most of the data gleaned from various classifiers can be used to lessen bias. This technique states that if one classifier is regarded as “expert”, aggregating the judgments of numerous “experts” will result in a more trustworthy outcome [114].

The three types of classification tasks that can be performed are binary, multiclass and multilabel.

A confusion matrix can be created by adding the outcomes of a binary classifier [115]. Metrics are typically produced based on combinations of values in the confusion matrix to reduce bias because each number represents a different facet of performance, and focusing on just one of them can introduce bias [116,117]. Additionally, the clinical context of the condition has a significant impact on the metrics of choice. High sensitivity is crucial, for instance, if a model is intended to screen for cancer; however, if the goal is to confirm cancer, a more specific model would be preferable. Setting up a relevant threshold for metrics is therefore crucial. If there is a significant imbalance in the data, relying on the “accuracy” statistic to assess model performance may result in bias. The ROC curve may more effectively illustrate model performance on uneven data than accuracy [118].

## 9. Conclusions

HNTs represent real challenges for clinicians and radiologists due to the complex regional anatomy, their often small sizes, the oncologic pathology variability, as well as the modifications of the anatomical site after treatment. Numerous promising studies have focused on radiomics and machine-learning applications for HNTs. While these techniques have the potential to overcome the current limitations of imaging in the head and neck area, future efforts must be directed toward robust external validation within multi-institutional collaborative efforts to standardize, refine and finally implement radiomics and machine-learning software in clinical practice.

## Figures and Tables

**Table 1:** Overview of study characteristics, divided by topic.

Article	Number of Patients	Subsite	Imaging	Analyzed Endpoint	Statistical Findings	Conclusion
Segmentation						
C. Parmar et al. Robust Radiomics Feature Quantification Using Semiautomatic Volumetric Segmentation [15]	20	Lung NSCLC	CT	Segmentation	56 3D radiomic features, quantifying phenotypic differences based on tumor intensity, shape and texture	Radiomic features extracted from 3D slicer segmentations had significantly higher reproducibility, were more robust and overlapping with the feature ranges extracted from manual contouring.
Kuhl, C.K.; Truhn, D. The Long Route to Standardized Radiomics: Unraveling the Knot from the End [16]	51	Soft-tissue sarcoma	CT, MRI and PET	Segmentation	169 preselected features	167 features demonstrated good to excellent reproducibility and 71 were reproducible after a comprehensive inter- and intra-CT image acquisition analysis.
Gitto, S. et al. Effects of Interobserver Variability on 2D and 3D CT- and MRI-Based Texture Feature Reproducibility of Cartilaginous Bone Tumors [17]	30	Bone tumors	CT and MRI	Segmentation	783 and 1132 features were extracted	The features extracted were reproducible. 3D and 2D MRI-based texture analyses provided similar rates of stable features.
Huan Yu et al. Coregistered FDG PET/CT-Based Textural Characterization of Head and Neck Cancer for Radiation Treatment Planning [18]	40	Head and neck cancer and lung cancer	F-FDG PET and CT	Segmentation	Texture features	Gray-tone difference matrices (NGTDM) (PET coarseness, PET contrast and CT coarseness) provided good discrimination performance.
Yu, H. et al. Automated Radiation Targeting in Head-and-Neck Cancer Using Region-Based Texture Analysis of PET and CT Images [19]	10	Head and neck cancer	F-FDG PET and CT	Segmentation	Co-registered multimodality pattern analysis segmentation system (COMPASS)	Tumor delineation was similar to those of the radiation oncologists.
Characterization						
Buch, K. et al. Using Texture Analysis to Determine Human Papillomavirus Status of Oropharyngeal Squamous Cell Carcinomas on CT [20]	40	Oropharyngeal carcinoma	CT	Characterization	A *t*-test evaluated differences in 42 texture features between HPV-positive and -negative carcinoma	There are statistically significant differences in some texture features between human-papillomavirus-positive and human-papillomavirus-negative oropharyngeal tumors.
Fujita, A et al. Difference Between HPV-Positive and HPV-Negative Non-Oropharyngeal Head and Neck Cancer [21]	46	Oral cavity, larynx and hypopharynx cancer	CT	Characterization	Texture analysis program extracted 42 texture features	16 texture parameters showed significant differences in relation to HPV status.
Vallieres, M. et al. FDG-PET Image-Derived Features Can Determine HPV Status in Head-and-Neck Cancer [22]	67	Hypopharynx	FDG-PET	Characterization	Six texture features, two SUV measures and three shape features were extracted, and logistic regression and support vector machine were performed	It is possible to predict HPV status and treatment failure in HNSCC using a combination of FDG-PET texture and morphological features.
Payabvash, S. et al. Differentiation of lymphomatous, metastatic, and non-malignant lymphadenopathy in the neck with quantitative diffusion-weighted imaging: Systematic review and meta-analysis [23]	Review (27 studies and 1165 patients)	Neck lymph nodes	MRI (Diffusion Weighted Imaging, DWI)	Characterization	Random-effects models, pooled diagnostic odds ratio (DOR), summary receiver operating characteristics (sROC), area under the curve (AUC) were determined	Quantitative valuation of ADC can help with differentiation of cervical lymph nodes. Lower ADC values are linked to malignancy and HPV positive status.
Payabvash, S. et al. Quantitative diffusion magnetic resonance imaging for prediction of human papillomavirus status in head and neck squamous-cell carcinoma: A systematic review and meta-analysis [24]	Review (5 studies and 264 patients)	HNSCC	MRI (DWI)	Characterization	Meta-analysis	HPV-positive HNSCC primary lesions have lower ADC.
Marzi, S.et al. Multifactorial Model Based on DWI-Radiomics to Determine HPV Status in Oropharyngeal Squamous Cell Carcinoma [25]	144	Oropharyngeal carcinoma	MRI (DWI)	Characterization	Different families of machine-learning (ML) algorithms and five-fold cross-validation	DWI-based radiomics can help in differentiating HPV-positive from HPV-negative patients.
Suh, C.H. et al. Oropharyngeal squamous cell carcinoma: Radiomic machine-learning classifiers from multiparametric MR images for determination of HPV infection status [26]	60	Oropharyngeal carcinoma	MRI	Characterization	1618 quantitative features extraction, features selection, three machine-learning classifiers (logistic regression, random forest and XG boost)	The highest diagnostic accuracies were achieved when using all sequences, and the difference was significant only when the combination did not include the ADC map.
Sohn, B. et al. Machine Learning Based Radiomic HPV Phenotyping of Oropharyngeal SCC: A Feasibility Study Using MRI [27]	62	Oropharyngeal carcinoma	MRI	Characterization	170 radiomic features	Six radiomic features with strong association with HPV status of SCC were selected using least absolute shrinkage and selection operator (LASSO).
Aerts, H.J.W.L. et al. Decoding tumour phenotype by noninvasive imaging using a quantitative radiomics approach [28]	1019	Lung or head-and-neck cancer	CT	Characterization	440 features	Some radiomic features had prognostic power associated with underlying gene expression patterns.
Zwirner, K. et al. Radiogenomics in head and neck cancer: Correlation of radiomic heterogeneity and somatic mutations in TP53, FAT1 and KMT2D [29]	20	HNSCC	CT	Characterization	Radiomic features and genetic analysis	Somatic mutations in *FAT1* and smaller primary tumor volumes were associated with reduced radiomic intra-tumor heterogeneity.
Huang, C. et al. Development and validation of radiomic signatures of head and neck squamous cell carcinoma molecular features and subtypes [30]	113	HNSCC	CT	Characterization	540 features, logistic regression, AUC	Quantitative image features can distinguish several molecular phenotypes.
Zhu, Y. et al. Imaging-Genomic Study of Head and Neck Squamous Cell Carcinoma: Associations Between Radiomic Phenotypes and Genomic Mechanisms via Integration of The Cancer Genome Atlas and The Cancer Imaging Archive [31]	126	HNSCC	CT	Characterization	Linear regression and gene set enrichment analysis	Associations between genomic features and radiomic features
Chen, R.-Y. et al.; Associations of Tumor PD-1 Ligands, Immunohistochemical Studies, and Textural Features in 18F-FDG PET in Squamous Cell Carcinoma of the Head and Neck [32]	53	HNSCC	18F-FDG PET	Characterization	Associations of tumor PD-1 ligands, immunohistochemical studies and textural features	PD-L1 expressions were positively correlated with *Ki-67 c-Met* and *p16.*
Brown, A.M. et al.; Multi-institutional validation of a novel textural analysis tool for preoperative stratification of suspected thyroid tumors on diffusion-weighted MRI [33]		Thyroid tumors	MRI (DWI)	Characterization	21 textural features	Textural analysis (TA) could characterize thyroid nodules using diffusion-weighted MRI (DW-MRI).
Jansen, J.F. Texture analysis on parametric maps derived from dynamic contrast-enhanced magnetic resonance imaging in head and neck cancer [34]	19	HNSCC	Dynamic contrast enhanced (DCE)-MRI	Characterization	Image texture analysis was employed on maps of K^trans^ and v_e_, generating two texture measures	Chemoradiation treatment in HNSCC significantly reduced the heterogeneity of tumors.
Kim, S. et al. Prediction of Response to Chemoradiation Therapy in Squamous Cell Carcinomas of the Head and Neck Using Dynamic Contrast-Enhanced MR Imaging [35]	33	HNSCC	DCE-MRI	Characterization	The data were analyzed by using SSM for estimation of K^trans^, v_e_ and τ_i_	Pretreatment DCE-MR imaging can potentially be used for prediction of response to chemoradiation therapy.
Shukla-Dave et al. Dynamic Contrast-Enhanced Magnetic Resonance Imaging as a Predictor of Outcome in Head-and-Neck Squamous Cell Carcinoma Patients with Nodal Metastases [36]	64	HNSCC	DCE-MRI	Characterization	DCE-MRI data were analyzed using the Tofts model	Important role of pretreatment DCE-MRI parameter K{sup trans} as a predictor of outcome
Dang, M. et al.; MRI Texture Analysis Predicts p53 Status in Head and Neck Squamous Cell Carcinoma [37]	16	HNSCC	MRI	Characterization	Texture analysis	MR imaging texture analysis predicted p53 status.
Staging						
Wang, F. et al. Radiomic Nomogram Improves Preoperative T Category Accuracy in Locally Advanced Laryngeal Carcinoma [38]	211	Laryngeal carcinoma	CT	Staging	1390 radiomic features extracted and analyzed	Eight features were found associated with preoperative T category.
Ren, J. et al.; Magnetic resonance imaging based radiomics signature for the preoperative discrimination of stage I-II and III-IV head and neck squamous cell carcinoma [39]	127	HNSCC	MRI	Staging	Radiomics signatures were constructed with least absolute shrinkage and selection operator (LASSO) logistic regression and analyzed	Radiomics signature based on MRI could discriminate stage I–II from stage III–IV HNSCC.
Romeo, V. et al. Prediction of Tumor Grade and Nodal Status in Oropharyngeal and Oral Cavity Squamous-cell Carcinoma Using a Radiomic Approach [40]	40	Oropharyngeal oral cavity carcinoma	CT	Staging	TA features	Tumor grade (TG) and nodal status (NS) could be predicted.
Wang, H. et al.; Machine learning-based multiparametric MRI radiomics for predicting the aggressiveness of papillary thyroid carcinoma [41]	120	Papillary thyroid carcinoma	MRI	Staging	1393 features	Aggressive and non-aggressive PTC could be distinguished preoperatively through machine-learning-based multiparametric MR imaging radiomics.

**Table 2:** Overview of study characteristics, divided by topic.

Article	Number of Patients	Subsite	Imaging	Analyzed Endpoint	Statistical Findings	Conclusion
Treatment						
Fave X et al. Delta-radiomics features for the prediction of patient outcomes in non-small cell lung cancer [42]	107	NSCC (lung)	CT	Overall survival, distant metastases and local recurrence	Multivariate models were built for overall survival, distant metastases and local recurrence using only clinical factors, clinical factors combined with pretreatment radiomics features, and a combination of clinical factors, pretreatment radiomics features and delta radiomics features	For overall survival and distant metastases, pretreatment compactness improved the c-index. For local recurrence, pretreatment imaging features were not prognostic, while texture strength measured at the end of treatment significantly stratified high- and low-risk patients.
Jansen JF et al. Texture analysis on parametric maps derived from dynamic contrast-enhanced magnetic resonance imaging in head and neck cancer [34]	19	HNSCC	CT and MRI	Prediction of treatment response	Image texture analysis was employed on maps of K^trans^ and V_e_, generating two texture measures: energy (E) and homogeneity	Chemoradiation treatment in HNSCC significantly reduced the heterogeneity of tumors.
Brown AM et al. Multi-institutional validation of a novel textural analysis tool for preoperative stratification of suspected thyroid tumors on diffusion-weighted MRI [33]	44	Thyroid cancer	MRI	Preoperative stratification	Apparent diffusion coefficients (ADCs) were obtained from regions of interest (ROIs) defined on thyroid nodules. TA, linear discriminant analysis (LDA) and feature reduction were also performed using the 21 MaZda-generated texture parameters that best distinguished benign and malignant ROIs	TA classified thyroid nodules with high sensitivity and specificity.
Zhang B et al. Radiomics Features of Multiparametric MRI as Novel Prognostic Factors in Advanced Nasopharyngeal Carcinoma [43]	118	Nasopharynx carcinoma	MRI	Progression-free survival (PFS)	A total of 970 radiomics features were extracted from T2-weighted (T2-w) and contrast-enhanced T1-weighted (CET1-w) MRI. Least absolute shrinkage and selection operator (LASSO) regression was applied to select features for progression-free survival (PFS) nomograms	Multiparametric MRI-based radiomics nomograms provided improved prognostic ability in advanced NPC.
Wang, G et al. Pretreatment MR imaging radiomics signatures for response prediction to induction chemotherapy in patients with nasopharyngeal carcinoma [44]	120	Nasopharynx carcinoma	MRI	Pretreatment prediction of early response to induction chemotherapy	Radiomics signatures were obtained with the least absolute shrinkage and selection operator method (LASSO) logistic regression model	Pretreatment morphological MR imaging radiomics signatures can predict early response to induction chemotherapy in patients with NPC.
Liu, J et al. Use of texture analysis based on contrast-enhanced MRI to predict treatment response to chemoradiotherapy in nasopharyngeal carcinoma [45]	53	Nasopharynx carcinoma	MRI	Pretreatment prediction of response to chemotherapy	Quantitative image parameters were extracted and statistically filtered to identify a subset of reproducible and non-redundant parameters, which were used to construct the predictive model. Internal validation was performed using stratified 10-fold cross-validation in the training set, and external validation was performed in the testing set. McNemar’s test was used to test the statistical difference between the performances of the extracted parameters in predicting the treatment response	Texture analysis based on T1 W, T2 W and DWI could act as imaging biomarkers of tumor response to chemoradiotherapy in NPC patients.
Romeo, V. et al. Prediction of Tumor Grade and Nodal Status in Oropharyngeal and Oral Cavity Squamous-cell Carcinoma Using a Radiomic Approach [40]	40	Oropharyngeal (OP) and oral cavity (OC) squamous-cell carcinoma (SCC)	CT	Prediction of tumor grade (TG) and nodal status (NS)	CT images were post-processed to extract TA features from primary tumor lesions. A feature selection method and different ML algorithms were applied to find the most accurate subset of features to predict TG and NS	A radiomic ML approach applied to PTLs was able to predict TG and NS in patients with OC and OP SCC.
Hawkins, P.G. et al. Sparing all salivary glands with IMRT for head and neck cancer: Longitudinal study of patient-reported xerostomia and head-and-neck quality of life [46]	252	HNSCC	Radiation Therapy	Prediction of xerostomia	Longitudinal regression was used to assess the relationship between questionnaire scores and mean bilateral parotid gland (bPG), contralateral submandibular gland (cSMG) and oral cavity (OC) doses. Marginal R^2^ and Akaike information criterion (AIC) were used for model evaluation	Reducing doses to all salivary glands maximized PROMs. A cSMG dose constraint of ≤39Gy did not increase failure risk.
Sheikh, K. et al. Predicting acute radiation induced xerostomia in head and neck Cancer using MR and CT Radiomics of parotid and submandibular glands [47]	266	HNSCC	CT and MRI	Prediction of xerostomia	CT and MR images were registered, on which glands were contoured. Image features were extracted for glands relative to the location of the primary tumor. Dose-volume-histogram (DVH) parameters were also acquired. Features were preselected based on Spearman correlation	Baseline CT and MR image features may reflect baseline salivary gland function and potential risk of radiation injury.
Liu, Y. et al. Early prediction of acute xerostomia during radiation therapy for nasopharyngeal cancer based on delta radiomics from CT images [48]	35	Nasopharynx cancer	CT	Prediction of xerostomia	RidgeCV and recursive feature elimination (RFE) were used for feature selection, while linear regression was used for predicting SA_30F_	Investigating radiation-induced changes of computed tomography (CT) radiomics in parotid glands (PGs) and saliva amount (SA) can predict acute xerostomia during the RT for nasopharyngeal cancer (NPC).
van Dijk, L.V. et al. Parotid gland fat related Magnetic Resonance image biomarkers improve prediction of late radiation-induced xerostomia [49]	68	HNSCC	MRI	Prediction of xerostomia	The performance of the resulting multivariable logistic regression models after bootstrapped forward selection was compared with that of the logistic regression reference model	Pretreatment MR-imaging biomarkers were associated with radiation-induced xerostomia, which supported the hypothesis that the amount of predisposed fat within the parotid glands is associated with Xer_12m_. In addition, xerostomia prediction was improved with MR-IBMs compared to the reference model.
van Dijk, L.V. et al. CT image biomarkers to improve patient-specific prediction of radiation-induced xerostomia and sticky saliva [50]	249	HNSCC	CT	Prediction of xerostomia	The potential IBMs represent geometric, CT intensity and textural characteristics of the parotid and submandibular glands. LASSO regularization was used to create multivariable logistic regression models, which were internally validated by bootstrapping	Prediction of XER_12m_ and STIC_12m_ was improved by including IBMs representing heterogeneity and density of the salivary glands, respectively. These IBMs could guide additional research into the patient-specific response of healthy tissue to radiation dose.
Thor, M. et al. A magnetic resonance imaging-based approach to quantify radiation-induced normal tissue injuries applied to trismus in head and neck cancer [51]	10	HNSCC	MRI	Prediction of trismus	Univariate logistic regression with bootstrapping (1000 populations) was applied to compare the muscle mean dose and textures between cases and controls (ipsilateral muscles); candidate predictors were suggested with an average *p* ≤ 0.20 across all bootstrap populations	TA identified the critical muscle(s) for radiation-induced trismus.
Abdollahi, H. et al. Cochlea CT radiomics predicts chemoradiotherapy induced sensorineural hearing loss in head and neck cancer patients: A machine learning and multi-variable modelling study [52]	47	HNSCC	CT	Prediction of sensorineural hearing loss	Different ML algorithms and LASSO logistic regression were implemented on radiomic features for feature selection, classification and prediction	A combination of radiomic features with clinical and dosimetric variables can model radiotherapy outcome, such as sensorineural hearing loss.
Metastases and Recurrence						
Kann, B.H. et al. Pretreatment Identification of Head and Neck Cancer Nodal Metastasis and Extranodal Extension Using Deep Learning Neural Networks [53]	270	HNSCC	CT	Identification of metastasis (nodal metastasis and tumor extranodal extension)	Three-dimensional convolutional neural network using a dataset of 2,875 CT-segmented lymph node samples with correlating pathology labels, cross-validated and tested on a blinded test set	The model has the potential for clinical decision making.
Kann, B.H. et al. Multi-Institutional Validation of Deep Learning for Pretreatment Identification of Extranodal Extension in Head and Neck Squamous Cell Carcinoma [54]	200 lymph nodes	HNSCC	CT	Identification of metastasis (extranodal extension ENE)	Deep-learning algorithm performance	Deep learning successfully identified ENE in pretreatment imaging.
Zhang, L. et al. Development and validation of a magnetic resonance imaging-based model for the prediction of distant metastasis before initial treatment of nasopharyngeal carcinoma: A retrospective cohort study [55]	176	Nasopharyngeal carcinoma	MRI	Identification of metastasis	Features of primary tumors were extracted; then, minimum redundancy–maximum relevance, LASSO and selection operator algorithms were performed. To select the strongest features, a logistic model for DM prediction was built	The model could be used as a prognostic model and can improve treatment decisions.
Bogowicz, M. et al. Computed Tomography Radiomics Predicts HPV Status and Local Tumor Control After Definitive Radiochemotherapy in Head and Neck Squamous Cell Carcinoma [56]	149	HNSCC	CT	Prediction of local tumor control (LC) after radiochemotherapy and HPV status	317 CT radiomic features were calculated. Cox and logistic regression models were built. The quality of the models was assessed using the concordance index (CI) for modeling of LC and receiver operating characteristics area under the curve (AUC)	Heterogeneity of HNSCC tumor density is associated with LC after radiochemotherapy and HPV status.
Li, S. et al. Use of Radiomics Combined With Machine Learning Method in the Recurrence Patterns After Intensity-Modulated Radiotherapy for Nasopharyngeal Carcinoma: A Preliminary Study [57]	306	Nasopharyngeal carcinoma	MRI, PET	Prediction of recurrence and radio resistance	1117 radiomic features were quantified from the tumor region intraclass correlation coefficients (ICC), and Pearson correlation coefficient (PCC) was calculated to identify the influential feature subset. Kruskal–Wallis test and receiver operating characteristic (ROC) analysis were employed to assess the ability of each feature in NPC-in-field recurrences prediction. Artificial neural network (ANN), k-nearest neighbor (KNN) and support vector machine (SVM) models were trained and validated by using stratified 10-fold cross-validation	In-field and high-dose region relapses were the main recurrence patterns, which may be due to the radioresistance. After integration with the clinical workflow, radiomic analyses can serve as imaging biomarkers to facilitate early salvage for NPC patients who are at risk of in-field recurrence.
Kuno, H. et al. CT Texture Analysis Potentially Predicts Local Failure in Head and Neck Squamous Cell Carcinoma Treated with Chemoradiotherapy [58]	62	HNSCC	CT	Prediction of local failure	Texture analysis	Independent primary tumor CT texture analysis features are linked to local failure after chemoradiotherapy in patients with HNSCC.
MDACC Head. Investigation of radiomic signatures for local recurrence using primary tumor texture analysis in oropharyngeal head and neck cancer patients [59]	465	Oropharyngeal cancer	CT, MRI, PET	Prediction of local recurrence	Two texture analysis features from pre-therapy imaging were extracted, and the resultant groups were analyzed	There is robust discrimination of recurrence probability and local control rate (LCR) differences between “favorable” and “unfavorable” clusters.
Zhang, L. et al. Radiomic Nomogram: Pretreatment Evaluation of Local Recurrence in Nasopharyngeal Carcinoma based on MR Imaging [60]	140	Nasopharyngeal carcinoma	MRI	Prediction of local recurrence	970 radiomic features were extracted. Univariate and multivariate analyses were used. Eight CET1-w image features and seven T2-w image features were selected to build a Cox proportional hazard model in the training cohort	This study demonstrates that MR-imaging-based radiomics can be used to categorize patients into low- and high-risk groups.
Survival						
Shen, H. et al. Predicting Progression-Free Survival Using MRI-Based Radiomics for patients with nonmetastatic Naso-pharyngeal Carcinoma [61]	327	Nasopharynx carcinoma	MRI	Prediction of progression-free survival (PFS)	The clinical and MRI data were collected. The least absolute shrinkage selection operator (LASSO) and recursive feature elimination (RFE) were used to select radiomic features. Five models were constructed. The prognostic performances of these models were evaluated by Harrell’s concordance index (C-index). The Kaplan–Meier method was applied for the survival analysis	The model incorporating radiomics, overall stage and Epstein–Barr virus DNA showed better performance in predicting PFS in non-metastatic NPC patients.
Yuan, Y. et al. MRI-based radiomic signature as predictive marker for patients with head and neck squamous cell carcinoma [62]	85	HNSCC	MRI	Prediction of prognosis	LASSO Cox regression model was used to select the most useful prognostic features with their coefficients, upon which a radiomic signature was generated	MRI-based radiomic signature is an independent prognostic factor for HNSCC patients.
Parmar, C. et al. Radiomic Machine-Learning Classifiers for Prognostic Biomarkers of Head and Neck Cancer [63]	196	HNSCC	CT	Prediction of overall survival	A total of 440 radiomic features were extracted from the segmented tumor regions in CT images. Feature selection and classification methods were compared using an unbiased evaluation framework	The study identified prognostic and reliable machine-learning methods for the prediction of overall survival of head and neck cancer patients.
Agarwal, J.P. et al. Tumor radiomic features complement clinico-radiological factors in predicting long-term local control and laryngectomy free survival in locally advanced laryngo-pharyngeal cancers [64]	60	Laryngopharynx cancer	CT	Prediction of long-term local control and laryngectomy-free survival (LFS)	The ability of texture analysis to predict LFS or local control was determined using Kaplan–Meier analysis and multivariate Cox model	Medium texture entropy is a predictor for inferior local control and laryngectomy-free survival in locally advanced laryngo-pharyngeal cancer, and this can complement clinico-radiological factors in predicting the prognosis of these tumors.
Liu, Z. et al. Radiomics-based prediction of survival in patients with head and neck squamous cell carcinoma based on pre- and post-treatment 18F-PET/CT [65]	171	HNSCC	PET-CT	Prediction of survival	Receiver operating characteristic (ROC) curves and decision curves were used to compare the predictions of ML models with those of a model incorporating only clinicopathological features	Combining clinicopathological characteristics with radiomics features of pre-treatment PET/CT or post-treatment PET/CT assessment of primary tumor sites as positive or negative may substantially improve the prediction of overall survival and disease-free survival of HNSCC patients.
Zhai, T.-T. et al. The prognostic value of CT-based image-biomarkers for head and neck cancer patients treated with definitive (chemo-)radiation [66]	444	HNSCC	CT	Prediction of local control (LC), regional control (RC), distant-metastasis-free survival (DMFS) and disease-free survival (DFS)	Models were created from multivariable Cox proportional hazard analyses based on clinical features and IBMs for LC, RC, DMFS and DFS	For prediction of HNC treatment outcomes, image biomarkers performed as well or slightly better than clinical variables.
Leijenaar, R.T.H. et al. External validation of a prognostic CT-based radiomic signature in oropharyngeal squamous cell carcinoma [67]	542	Oropharyngeal carcinoma	CT	Prognosis prediction	Signature model was tested and fit in a Cox regression and assessed model discrimination with Harrell’s c-index. Kaplan–Meier survival curves between high and low signature predictions were compared with a log-rank test	Signature had significant prognostic power, regardless of whether patients with CT artifacts were included.
Liu, J. et al. Use of texture analysis based on contrast-enhanced MRI to predict treatment response to chemoradiotherapy in nasopharyngeal carcinoma [45]	53	Nasopharyngeal carcinoma	MRI	Treatment prediction	Quantitative image parameters were extracted and statistically filtered to identify a subset of reproducible and non-redundant parameters, which were used to construct the predictive model. McNemar’s test was used to test the statistical difference in predicting the treatment response	Texture analyses based on T1 W, T2 W and DWI could act as imaging biomarkers of tumor response to chemoradiotherapy in NPC patients and serve as a new radiological analysis tool for treatment prediction.
Bogowicz, M. et al. Perfusion CT radiomics as potential prognostic biomarker in head and neck squamous cell carcinoma [68]	45	HNSCC	CT perfusion (CTP)	Prediction of local tumor control	Each feature was assigned to a principal component group based on feature–principal component correlation. Univariate Cox regression analysis was used to define the best prognostic feature in each group	CTP radiomics is a prognostic factor for local tumor control after definitive radiochemotherapy.
Zhang, H. et al. Locally Advanced Squamous Cell Carcinoma of the Head and Neck: CT Texture and Histogram Analysis Allow Independent Prediction of Overall Survival in Patients Treated with Induction Chemotherapy [69]	72	HNSCC	CT	Prediction of overall survival	CT texture and histogram analyses of primary mass on pretherapy CT images were performed by using TexRAD software before and after application of spatial filters at different anatomic scales, ranging from fine detail to coarse features. Cox proportional hazards models were used to examine the association between overall survival and the baseline CT imaging measurements and clinical variables	Independent of tumor size, N stage and other clinical variables, primary mass CT texture and histogram analysis parameters were associated with overall survival in patients with locally advanced squamous cell carcinoma of the head and neck who were treated with induction TPF.
Mao, J. et al. Predictive value of pretreatment MRI texture analysis in patients with primary nasopharyngeal carcinoma [70]	79	Nasopharyngeal carcinoma	MRI	Prediction of progression-free survival (PFS)	The Cox proportional hazards model was used to determine the association of texture features, tumor volume and the tumor node metastasis (TNM) stage with PFS. Survival curves were plotted using the Kaplan–Meier method. The prognostic performance was evaluated with the receiver operating characteristic (ROC) analyses and C-index	A texture parameter of pretreatment CE-T1WI-based uniformity improved the prediction of PFS in NPC patients.
Cheng, N.-M. et al. Textural Features of Pretreatment 18 F-FDG PET/CT Images: Prognostic Significance in Patients with Advanced T-Stage Oropharyngeal Squamous Cell Carcinoma [71]	70	Oropharyngeal carcinoma	PET-CT	Prediction of prognosis	Uniformity extracted from the normalized gray-level co-occurrence matrix represents an independent prognostic predictor in patients with advanced T-stage OPSCC	Uniformity extracted from the normalized gray-level co-occurrence matrix represents an independent prognostic predictor in patients with advanced T-stage OPSCC.
Park, V.Y. et al. Association Between Radiomics Signature and Disease-Free Survival in Conventional Papillary Thyroid Carcinoma [72]	768	Thyroid carcinoma	Ultrasound	Identification of biomarkers for risk stratification	A radiomics signature (Rad-score) was generated by using the least absolute shrinkage and selection operator (LASSO) method in Cox regression	Radiomics features from pretreatment US may be potential imaging biomarkers for risk stratification in patients with conventional papillary carcinoma.
Zdilar, L. et al. Evaluating the Effect of Right-Censored End Point Transformation for Radiomic Feature Selection of Data From Patients With Oropharyngeal Cancer [73]	529	Oropharyngeal carcinoma	-	Prediction of overall survival (OS) and relapse-free survival (RFS)	Radiomic signatures combined with clinical variables were used for risk prediction. Three metrics for accuracy and calibration were used to evaluate eight feature selectors and six predictive models	Random regression forest and random survival forest performed best for OS and RFS, respectively.
Zhuo, E.-H. et al. Radiomics on multi-modalities MR sequences can subtype patients with non-metastatic nasopharyngeal carcinoma (NPC) into distinct survival subgroups [74]	658	Nasopharyngeal carcinoma	MRI	Revelation of distinct survival subtypes	Each patient in the validation cohort was assigned to the risk model using the trained classifier. Harrell’s concordance index (C-index) was used to measure the prognosis performance, and differences between subgroups were compared using the log-rank test	Quantitative multi-modalities MRI image phenotypes revealed distinct survival subtypes.
Haider, S.P. et al. Potential Added Value of PET/CT Radiomics for Survival Prognostication beyond AJCC 8th Edition Staging in Oropharyngeal Squamous Cell Carcinoma [75]	311	Oropharyngeal carcinoma	CT/ PET	Definition of staging scheme for survival prognostication and risk stratification	Harrell’s C-index quantified survival model performance; risk stratification was evaluated in Kaplan–Meier analysis	Radiomics imaging features extracted from pretreatment PET/CT may provide complementary information to the current American Joint Committee on Cancer staging scheme for survival prognostication and risk stratification of HPV-associated OPSCC.
Leijenaar, R.T. et al. Development and validation of a radiomic signature to predict HPV (p16) status from standard CT imaging: A multicenter study [76]	778	Oropharyngeal carcinoma	CT	Identification of the HPV status (p16) of OPSCC and prognosis	Multivariable modeling was performed using least absolute shrinkage and selection operator. Kaplan–Meier survival analysis was performed to compare HPV status based on p16 and radiomic model predictions	Radiomics has the potential to identify clinically relevant molecular phenotypes influencing the prognosis.
